# Using a Simple Apparatus to Measure Direct and Diffuse Photosynthetically Active Radiation at Remote Locations

**DOI:** 10.1371/journal.pone.0115633

**Published:** 2015-02-10

**Authors:** Michael J. Cruse, Christopher J. Kucharik, John M. Norman

**Affiliations:** 1 Department of Agronomy, University of Wisconsin—Madison, 1575 Linden Drive, Madison, WI, 53706, United States of America, Nelson Institute for Environmental Studies, University of Wisconsin—Madison, 550 North Park St., Madison, WI, 53706, United States of America; 2 Department of Soil Science, University of Wisconsin—Madison, 1525 Observatory Drive, Madison, WI, 53706, United States of America; CSIRO, AUSTRALIA

## Abstract

Plant canopy interception of photosynthetically active radiation (PAR) drives carbon dioxide (CO_2_), water and energy cycling in the soil-plant-atmosphere system. Quantifying intercepted PAR requires accurate measurements of total incident PAR above canopies and direct beam and diffuse PAR components. While some regional data sets include these data, e.g. from Atmospheric Radiation Measurement (ARM) Program sites, they are not often applicable to local research sites because of the variable nature (spatial and temporal) of environmental variables that influence incoming PAR. Currently available instrumentation that measures diffuse and direct beam radiation separately can be cost prohibitive and require frequent adjustments. Alternatively, generalized empirical relationships that relate atmospheric variables and radiation components can be used but require assumptions that increase the potential for error. Our goal here was to construct and test a cheaper, highly portable instrument alternative that could be used at remote field sites to measure total, diffuse and direct beam PAR for extended time periods without supervision. The apparatus tested here uses a fabricated, solar powered rotating shadowband and other commercially available parts to collect continuous hourly PAR data. Measurements of total incident PAR had nearly a one-to-one relationship with total incident radiation measurements taken at the same research site by an unobstructed point quantum sensor. Additionally, measurements of diffuse PAR compared favorably with modeled estimates from previously published data, but displayed significant differences that were attributed to the important influence of rapidly changing local environmental conditions. The cost of the system is about 50% less than comparable commercially available systems that require periodic, but not continual adjustments. Overall, the data produced using this apparatus indicates that this instrumentation has the potential to support ecological research via a relatively inexpensive method to collect continuous measurements of total, direct beam and diffuse PAR in remote locations.

## Introduction

Measurements of total incoming PAR (PAR_T_) and its components, direct beam PAR (PAR_B_) and diffuse PAR (PAR_D_), at the site level are critical to field research investigating the exchange of CO_2_, water, and energy in the planetary soil-plant-atmosphere system. Besides their importance to individual research efforts, they contribute to the development of generalized relationships found within process-based ecosystem models that are often used across large spatial scales. These PAR data also provide critical model validation and driver data, helping to increase confidence in ecosystem models that are used to investigate the impacts of changing climate, atmospheric chemistry, and land use on regional to global scale biogeochemical cycling and net primary production [1, 2]. While simple and relatively inexpensive instrumentation is available to measure PAR_T_ at a field site, partitioning PAR_T_ into PAR_B_ and PAR_D_ is more difficult, forcing researchers to choose one of two paths. One option is to invest in more costly commercially available equipment, or a sometimes less desirable choice of using empirical equations that have reduced accuracy at the site level due to assumptions in model development and variable conditions across both space and time.

The measurement of above canopy PAR_T_, PAR_D_ and PAR_B_ is central to accurate simulation of canopy interception and absorption of PAR, processes that drive rates of canopy photosynthesis [3, 4]. The importance in separating PAR_B_ and PAR_D_ comes from the difference in attenuation of these two types of radiation through plant canopies, with PAR_B_ having a solar zenith angle dependence and PAR_D_ having no dependence [5, 6]. This leads to varying intensities of leaf level radiation loads as a function of canopy depth and structure. Accounting for the percentage of leaf level radiation that is diffuse and direct beam is also important because overall radiation loads lead to changes in photosynthetic rates and canopies can use PAR_D_ and PAR_B_ at different levels of efficiency [7]. For example, some plants are well adapted to diffuse PAR_D_ and can have leaf-level rates of photosynthesis under shaded conditions that are comparable to leaves exposed to full sun [8]. At the canopy level, an increase in diffuse radiation can lead to reduced annual carbon uptake by ecosystems because of the associated drop in radiation input [9]. Common modeling and scaling approaches account for these differences in attenuation and plant use of PAR_D_ and PAR_B_ by splitting the canopy into sunlit and shaded fractions, allowing for more accurate representation of radiation effects on photosynthesis [5, 10]. While the calculation of PAR_B_ and PAR_D_ at different canopy levels will depend on LAI, foliage distribution (e.g., clumping), optical properties, and solar zenith angles, the accuracy is largely dependent on quantifying incoming PAR_T_, PAR_B_ and PAR_D_ at the top of a canopy.

There are currently three categories of commercially available devices that collect continuous measurements of diffuse and direct beam radiation: sun tracking systems, stationary sun shades, and moving shadow bands. Examples of sun tracking systems include the Kipp and Zonen 2AP (Kipp & Zonen, The Netherlands) and the Eppley SMT-3 (The Eppley Laboratory, Inc. Newport, RI). Both of these systems use computer aided motorized systems to place shading discs in the direct path between the sun and the radiation sensor of choice. While these systems are very effective at what they do, at a cost of greater than $15,000 USD, they may prove prohibitive for some research projects. Products that include stationary shading devices are the Eppley SBS shadow band (The Eppley Laboratory, Inc. Newport, RI) and the SPN1 sunshine pyranometer (Delta-T Devices Ltd. Cambridge, United Kingdom). The SBS shade band uses a single shadowband positioned above a radiation sensor so that direct beam radiation is blocked continuously over the course of the day. These shade bands require accurate construction and regular adjustments to guarantee proper diffuse measurements, and cost over $5,000 USD when including the purchase of sensors and logging devices. The SPN1 sunshine pyranometer takes a unique approach by using multiple sensors and a stationary shading structure that ensures one of the sensors will be continuously shaded while another sensor will remain illuminated by full sunlight. This setup, costing around $9,000 USD including the sensor and data logger, requires that all system sensors produce consistent readings and that a glass dome be heated to remove dew. Irradiance, Inc. (Lincoln, MA) produces a moving shadowband system that periodically blocks direct radiation and can measure direct, diffuse and total incident radiation at a starting price of $8,750 USD.

In place of using commercially available equipment at individual sites, empirically derived models, many of which have evolved from the work of Liu and Jordan [11], are used to predict incident diffuse radiation at the earth’s surface. These empirical models are based upon the observed relationship between the diffuse fraction of global radiation (k_d_, ratio of the diffuse-to-global solar radiation) and an atmospheric clearness index (k_t_, ratio of the global-to-extraterrestrial solar radiation) [12]. These approaches typically use a piecewise data fitting exercise that produces three or more separate equations to calculate diffuse radiation as a function of k_t_. The equations were developed given notable differences in the relationship of k_t_ to diffuse radiation. Specifically, (1) at low k_t_ values there is a relatively high percentage of diffuse radiation, (2) at high k_t_ values, the majority of radiation is direct beam with a minimum percentage of diffuse that must be considered, and (3) between the two extremes there is a decline in the percentage of diffuse as k_t_ increases.

Additional variables have been incorporated into these models to help improve estimates of diffuse radiation [13]. In addition to equations being dependent on solar zenith angle and k_t_, they are often parameterized as a function of air temperature and relative humidity. Incoming radiation drives much of the diurnal pattern in surface air temperatures. Using measurements of the Bowen ratio, Bristow and Campbell [14] showed that there is a strong empirical relationship between the transmittance of the atmosphere, a variable that influences diffuse radiation levels, and the daily range in temperature. Water vapor is a major player in atmospheric radiation dynamics, both in interception and emittance [15], so including this quantity is expected to improve estimates of radiation attenuation. The inclusion of solar zenith angle is important because the further away from nadir the solar beam becomes, the longer the atmospheric path length that is required for the beam to reach the earth’s surface and the greater opportunity for radiation scattering [5].

While these approaches capture well the general principles of atmospheric radiation attenuation, their use can be limited by a number of factors. Most of the theories are empirically derived and have some degree of location dependence [13], requiring incorporation of site specific characteristics and calibration before use of such equations. A list of important site characteristics that would affect the accuracy of these approaches include latitude and longitude, prevailing atmospheric conditions at the site (e.g. primarily a foggy area, little to no cloud cover, etc.), proximity to anthropogenic disturbances that would influence the atmosphere (e.g. power plants, forest fires) as well as how the average site conditions influence model parameterization and calibration. The theoretical approaches are also somewhat limited by the use of piecewise data fitting strategies and the assumptions that are used to restrict those data fitting procedures.

Given the expense of available diffuse radiation measurement devices and the complications inherent to empirical relationships used to predict incident diffuse radiation levels, the objectives of this project were twofold: (1) to construct a simple PAR measuring device that can capture PAR_T_ into PAR_B_ and PAR_D_ components without requiring periodic shadowband adjustments; (2) to evaluate the instrument by comparing data to the expected seasonal and diurnal behavior of diffuse and direct beam PAR expressed by models, and to illustrate temporal PAR_B_ and PAR_D_ variability that is unable to be captured by generalized empirical relationships. Our overarching goal is to demonstrate that a simple PAR measurement apparatus can collect robust data at field sites that is at a lower cost than other commercially available equipment and provides superior data compared to those derived from previously published empirical models.

Disclaimer: We the authors do not endorse or discourage the purchase or use of equipment from companies described in this article. Products described here are meant to serve as examples for setup of the described system and could be adjusted with other available equipment at the reader’s discretion.

## Material and Methods

### 2.1 System design

The general concept of the system we constructed and tested in this study is similar to those described by Wesely [16] and Michalsky et al. [17] as well as the commercially-available product sold by Irradiance, Inc., where a single radiation sensor is routinely shaded by a moving shadowband providing measurements of incoming radiation. In our case, we are specifically producing measurements of PAR_T_, PAR_B_ and PAR_D_ (All symbols are described in Table 1). Measurements of PAR were collected using a LI-190 point quantum sensor (LI-COR Inc., Lincoln, NE). The LI-190 is a silicon photodiode based radiation sensor that makes close to optimal measurements of incident radiation between 400 and 700 nm under most environmental conditions with the aid of colored glass and interference filters [18]. The sensor has an attached millivolt adapter with bare wire connections that allows for connection to the datalogger (CR1000, Campbell Scientific, Inc., Logan, UT), which collects readings from the sensor every second and records data at intervals described later in the theory of measurement section. The LI-190 is installed on a mounting base (2003S, LI-COR Inc., Lincoln, NE) that allows for leveling.

**Table 1:** Definition of symbols.

**Symbol**	**Description**	**Units**	**Equation**
PAR_T_	Total incident PAR	μmol photons m^-2^ s^-1^	1, 3
PAR_B_	Direct beam component of PAR_T_	μmol photons m^-2^ s^-1^	3
PAR_D_	Diffuse component of PAR_T_	μmol photons m^-2^ s^-1^	1, 3
PAR_M_	Average reading measured by the shadowband system	μmol photons m^-2^ s^-1^	1
t_mean_	Time interval that averaging is performed over	Minutes	1
t_D_	The total time the sensor is covered by the shadowband during one rotation	Minutes	1, 2
N	Number of times the shadowband passes over the radiation sensor during t_mean_		1
S_w_	Width of the shadowband	cm	2
S_r_	Radius of the shadowband arc	cm	2
ω	The rotational distance that the shadowband revolves in one minute	degrees	2
k_t_	Clearness index; ratio of the global-to-extraterrestrial solar radiation		
k_d_	Ratio of the diffuse-to-global solar radiation		
R	Parameter in the regression of diffuse share on transmission		
K	Parameter in the regression of diffuse share on transmission		
β	Solar elevation angle		

**Figure 1:**
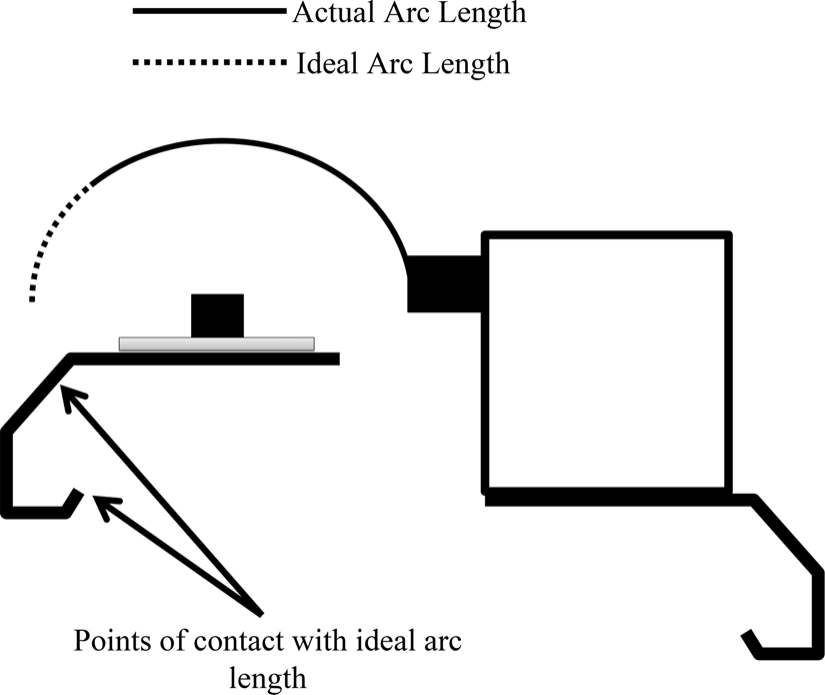
Difference between ideal arch length and actual, with points of contact that would not allow the use of the ideal arc length.

The shadowband revolves completely and continuously around the LI-190 at a rate of 12 revolutions per hour, and shades or partially obstructs the view of the LI-190 only when passing above the sensor surface area. The complete shading of the radiation sensor blocks all of the direct beam radiation and provides a measurement of incident diffuse radiation. The shadowband itself is a fabricated piece of stainless steel painted dull black with a width (S_w_) of 2 cm and bent in an arc with a consistent radius (S_r_) of 4.2 cm between the shadowband and sensor. The ratio of S_w_ to S_r_ (0.476) is higher than other shadowband designs [19] which is important when considering corrections that must be applied post data collection. The shadowband arc is 19 cm long and provides coverage of all but 30 degrees on the end of the arc not connected to and opposite of the motor. The arc could not cover all view angles because a shorter arc length was required to allow passage under the radiation mount stand (CM225, Campbell Scientific, Inc., Logan, UT, Fig. 1) but the arc does block all solar zenith angles that occur during the growing season at the research site (43°17’44” N, 89°22’48” W). To maximize arc length, the CM225 was modified by cutting out two sections, approximately 2.5 cm by 2.5 cm, on the mounting arm. The shadowband movement is powered by an AC synchronous timing motor (H1–29, Herbach and Rademan, Inc., Moorestown, NJ), allowing for the greatest control of the revolution speed of the shadowband. The shadowband is connected to the motor’s rotating shaft by a cylindrical piece of solid aluminum (radius of 0.75 cm and length of 2.5 cm) with a central shaft bored out for a set screw and two screw taps to allow connection to the shadowband. The motor itself is mounted on another CM225 and housed in a fabricated stainless steel box to protect it from weather elements. All components that are at or above the sensor height at any time are painted with a dull black finish.

The presented setup is for remote locations, with no source of AC power, and so the only available source of continuous power for the system is a DC rechargeable battery. To power the AC motor (with a maximum current draw of 4 Watts at 110 VAC, 60 Hz) consistently, a pure sine wave inverter (GP-SW150–12, Carmanah, British Columbia, Canada) is required, which when coupled with the motor raises the continuous power consumption of the system to 21.6 Ah. This consumption level requires maximum performance and recharging of a 24 Ah battery. Therefore, to guarantee consistent operation for this power draw, a rechargeable power supply was needed that includes a high-capacity 84 Ahr rechargeable battery (BP84, Campbell Scientific, Inc., Logan, UT), regulator (CH100-SW, Campbell Scientific, Inc., Logan, UT) and 70-watt solar panel (SP70, Campbell Scientific, Inc., Logan, UT). This system was also used in conjunction with data logger program commands that shut off the power supply to the motor during darkness. This programming is accomplished by routing the power supply for the motor through the 12 volt unregulated switch terminal included on the CR1000 datalogger and making proper additions to the programming language. Availability of an onsite AC power source would alleviate the need of the solar panel and inverter and further reduce the cost of the system.

**Figure 2:**
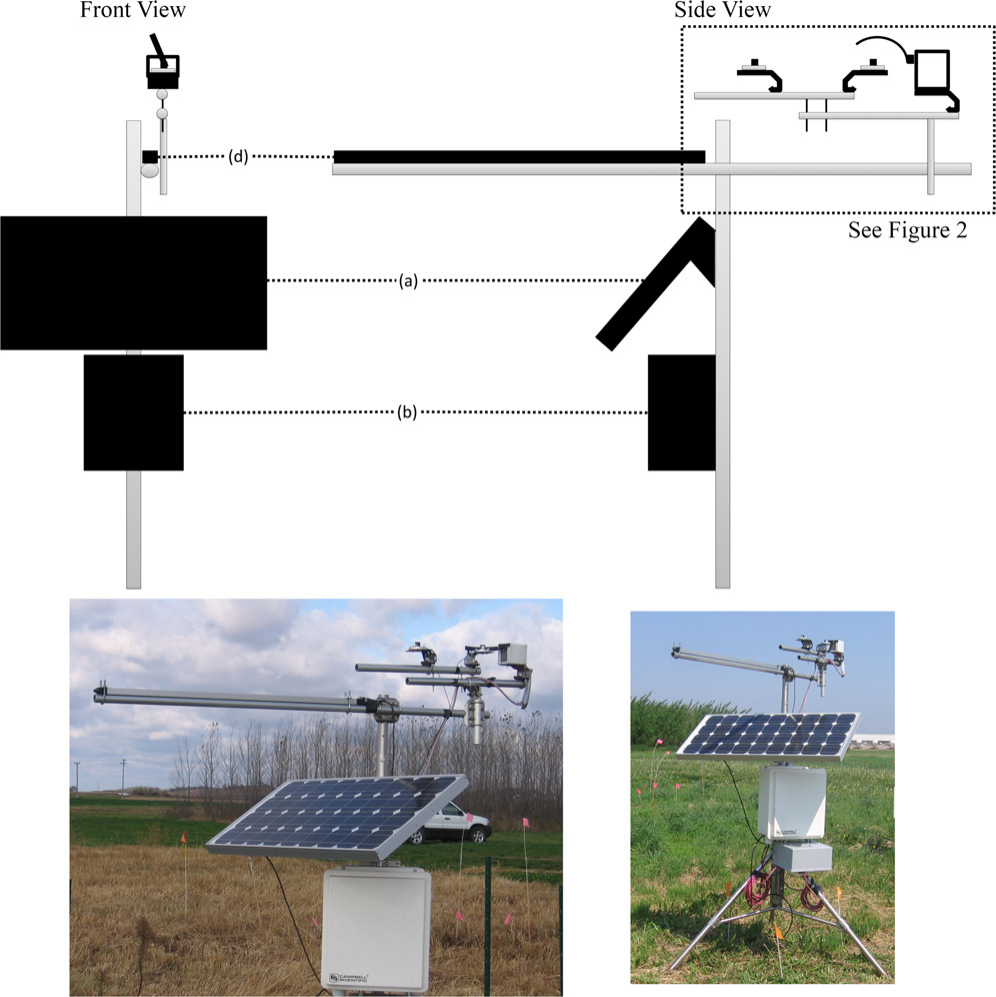
Large scale view of shadowband apparatus design (not to scale). Components listed in Table 2.

**Figure 3:**
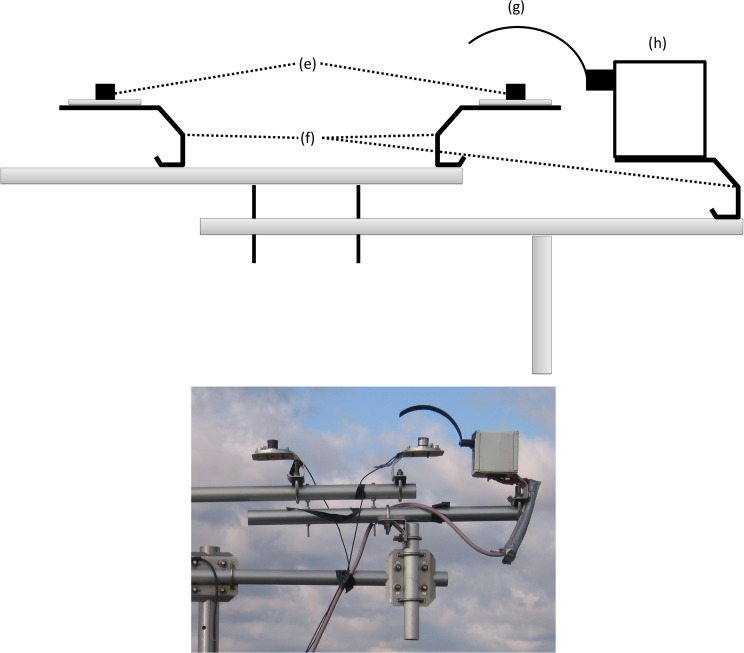
Side view zoom of shadowband apparatus design (not to scale). Components listed in Table 2.

All components are mounted using a combination of tripods, enclosures, crossbars and sensor stands readily available from Campbell Scientific, Inc. or other companies (Table 2). The radiation sensor was mounted slightly above 3 m at the research site because the sensor should only be shaded by the shadowband and the height of mounting needed to be sufficient enough to prevent shading from undesirable sources (e.g. buildings, plants). The shadowband design shown is used in an experiment investigating plant canopy and radiation interactions, so there are extra sensors that are not necessary for the measurement of PAR_T_, PAR_D_, and PAR_B_ (Figs. 2 and 3 and Table 2; 1 LI-190 and 1 LI-191, LI-COR Inc., Lincoln, NE).

**Table 2:** List of parts for shadowband system.

**Component**	**Figs. 1 and 2**	**Manufacturer: Part Number**
Tripod and crossarms		Campbell Scientific: CM110, CM206, CM202
Solar panel	a	Campbell Scientific: SP70
Regulator		Campbell Scientific: CH100-SW
Data logger		Campbell Scientific: CR1000
Battery		Campbell Scientific: BP84
Inverter		Carmanah: GP-SW150–12
Motor		Herbach and Rademan: H1–29
Sensor mounts	f	LI-COR: 2003S
Quantum sensors		
Point (2)	e	LI-COR: LI-190
Line (1)	d	LI-COR: LI-191
Shadowband	g	Fabricated
Motor box	h	Fabricated
Data logger housing	b	Campbell Scientific: ENC16/18

### 2.2 Shadowband theory

The theory explaining how the shadowband system measures direct and diffuse radiation components follows the work of Wesely [16] and Michalsky et al. [17] where the shading of the radiation sensor provides readings of PAR_D_ and PAR_T_, while PAR_B_ readings occur when the sensor is not covered. A primary difference between the theory presented in the aforementioned publications and the system described here is that direct measurements of PAR_T_ are not obtained by our instrument. Instead, our methodology uses weighted average functions accounting for the time the sensor is completely blocked versus not 100% covered by the shadowband to calculate PAR_T_. Therefore, measurements are not dependent on knowledge of where the shadowband is continuously located, which reduces measurement complexity.

With the shadowband revolving at a constant rate (e.g., 12 rev/hour), both minimum and average radiation readings can be captured over the course of an hour. The minimums equate to the period when the sensor is fully shaded from the sun’s direct beam by the shadowband, and provide a measurement of diffuse radiation (PAR_D_). The PAR_D_ value is an hourly average of the 12 minimums that are recorded when the shadowband completely obstructs the LI-190 quantum sensor. The average PAR reading during the entire measurement period (PAR_M_) is also calculated and is influenced by both shaded and non-shaded conditions. The PAR_M_ and PAR_D_ values can then be used to calculate total PAR (PAR_T_) using a simple weighted average equation.

$$PAR_{T}=\frac{PAR_{M}*t_{mean}-PAR_{D}*(N*t_{D})}{t_{mean}-(N*t_{D})}$$ (1)

In Equation 1, t_D_ is the total time in minutes that the PAR sensor is covered by the shadowband during one rotation (Equation 2), t_mean_ is the time interval in minutes that averaging is done over (e.g., 60 minutes) and N is the number of revolutions the shadow band makes over t_mean_.

$$t_{D}=\left(\frac{\frac{S_{w}}{S_{r}}*57.296}{\omega }\right)$$ (2)

In Equation 2, 57.296 converts radians to degrees and ω is the degrees that the shadowband revolves in one minute.

The PAR_D_ values are also corrected for the portion of the incoming diffuse PAR that is blocked by the shadowband during diffuse measurements. For stationary shading devices, corrections of this nature need to account for both the physical area blocked by the shadow device as well as the fact that the sky is anisotropic in nature and one view angle is consistently blocked by the stationary device [19]. Given that the current design does not block the same portion of view for any extended period of time and because all readings influenced by the shadowband are incorporated into PAR_M_, it was determined that applying a correction to PAR_D_ only was most appropriate. This correction to PAR_D_ should take into account the average interception of radiation by the rotating shadowband when measured over the course of the shadowband rotation arc. This correction is dependent upon both S_w_ and S_r_ and was determined to be 15% by collecting data under overcast conditions and comparing unobstructed sensor readings to readings taken with the band in multiple positions along its rotation path.

The final component of incoming PAR that can be determined is the direct beam portion (PAR_B_) and it is related to PAR_T_ and PAR_D_ through Equation 3 [20].

$$PAR_{B}=PAR_{T}-PAR_{D}$$ (3)

### 2.3 System deployment and site description

The system was deployed at the agronomic intensive site of the Great Lakes Bioenergy Research Center (GLBRC) at the Arlington Research Station, Arlington, Wisconsin (43°17’44” N, 89°22’48” W) during the 2011 and 2012 growing seasons. The site has a humid continental climate [21] with percentages of cloudy, partly cloudy, and clear days of 50%, 26% and 24%, respectively [22]. Mean annual air temperatures are 6.8°C and a mean annual rainfall is 869 mm (NOAA, 1981–2010). To prevent shading from the box enclosing the shadowband motor, the system was oriented so all components that were above the surface height of the LI-190 were orientated due north of the quantum sensor. If the system was used in the southern hemisphere, these same components should be orientated south of the quantum sensor.

### 2.4 Data Quality Assurance

A challenge when assessing data quality is the lack of a clear indicator in the recorded data that shows when the shadowband motor has enough power to operate. This is a potential problem when the DC power supply might be limited or intermittent, but with a stable AC power source or the solar panel and battery system described here, this should be a non-issue.

Additional steps that were taken to assure the use of quality data include:

- Hourly data was removed when zenith angles are above 80° due to measurement errors that occur with the cosine response of the LI-190 quantum sensor.
- Hourly data was removed when any of the calculated totals (PAR_T_, PAR_D_, or PAR_B_) are negative.
- All data was inspected visually to account for outlier data that does not realistically represent known weather conditions (e.g. radiation measurements that were too high for overcast days).
- If a second quantum sensor is available, as was for this study, all hourly data that has PAR_M_ values larger than the measurement of total PAR made by the unobstructed quantum sensor on the system was discarded.

After removing data when solar zenith angles were above 80°, the last three bullet points required the removal of about 19% of the potential data. To achieve greater confidence in data measured by a system similar to this the data logger programming could be adjusted to track system available voltage and a position sensor could be added to keep track of the changes in the shadowband position.

### 2.5 Verification

While the theory behind the measurements obtained is based on simple weighted average equations that are used along with previously published approaches outlined by Wesely [16] and Michalsky et al. [17], two data sources/methods were used to investigate the validity of data collected by the instrument constructed in this study. Calculated PAR_T_ values from the shadowband system were compared directly with total PAR values measured at the same location with other sensors and measured PAR_D_ values were compared with theoretical values that would be calculated if there was no diffuse measurement device available.

**2.5.1 Total PAR.** Given that the PAR_T_ values found using the shadowband system are dependent upon an accurate measurement of PAR_D_ and PAR_M_, the comparison of PAR_T_ from the shadowband system with other PAR_T_ measurements from the same locality can help evaluate Equation 1 and the accuracy of the PAR_D_ measurements. The system setup presented here contains an additional point quantum sensor (LI-190, LI-COR Inc., Lincoln, NE). Because these additional measurements have no influence on the calculations of PAR_T_ and are located at the same site as the shadowband system, they are ideal for comparison purposes.

**2.5.2 Diffuse Radiation.** Three previously published models were selected to compare with calculated PAR_D_ values (Table 3). To use additional empirical approaches, other values need to be quantified including k_t_, extraterrestrial radiation, and solar zenith angles. Calculations of extraterrestrial radiation were made using the approach of Spitters et al. [23], and solar zenith angles were calculated using methods from Campbell and Norman [5]. The atmospheric clearness index was calculated using the computed extraterrestrial radiation values as well as total solar radiation measured by a full spectrum pyranometer (LI-200, LI-COR, Inc.) located at the Arlington Agricultural Research Station, Arlington, WI. The LI-200 has a poor spectral response compared to other full spectrum pyranometers [18], especially under non-ideal conditions. Environmental conditions, such as air temperature and relative humidity, have been shown to have varying effects on the data collected by this instrument [24]. While corrections for these measurements can be found [25], the formula presented are usually empirical in nature, with coefficients dependent upon site specific conditions. We concluded that attempting to correct LI-200 measurements would induce more error into the data because there is uncertainty in the empirical corrections offered. Furthermore, measurements were only collected during the growing season, which exposed the LI-200 to a reduced temperature range, minimizing the impact on measurement accuracy.

**Table 3:** Empirical formulas.

**Source**	**Formula**	**Range**
Spitters et al., 1986	k_d_ = 1	k_t_ ≤ 0.22
	k_d_ = 1−6.4(k_t_−0.22)^2^	0.22 < k_t_ ≤ 0.35
	k_d_ = 1.47−1.66(k_t_)	0.35 < k_t_ ≤ K
	k_d_ = R	K < k_t_
	K = (1.47 −R)/1.66	
	R = 0.847−1.61*sinβ* + 1.04*sin^2^β*	
		
Erbs et al., 1982	k_d_ = 1−0.09(k_t_)	k_t_ ≤ 0.22
	k_d_ = 0.9511−0.1604(k_t_) + 4.388(k_t_)^2^−16.638(k_t_)^3^ + 12.336(k_t_)^4^	0.22 < k_t_ ≤ 0.8
	k_d_ = 0.165	0.8 < k_t_
		
Jacovides et al., 2010	k_d_ = 0.98	k_t_ ≤ 0.06
	k_d_ = 0.97 + 0.256(k_t_)− 3.33(k_t_)^2^ + 2.42(k_t_)^3^	0.06 < k_t_ ≤ 0.86
	k_d_ = 0.276	0.86 < k_t_

**2.5.3 Statistical Analysis**. Statistical analysis was performed on a pooled data set of hourly averaged data from daylight hours measured across the growing seasons of 2011 and 2012. Analysis of measured and predicted percentages of diffuse incident radiation was completed using paired t-test and analysis of hourly averaged radiation data was performed using Deming regression techniques using a predesigned macro [26] in SAS 9.4 statistical software (SAS Institute Inc.). The final data set contained over 3300 hours for comparative analysis. For the t-tests values were considered significant at P < 0.05. Slopes, intercepts and confidence intervals were used to assess divergence from ideal one-to-one relationships.

## Results

### 3.1 Total PAR and Diffuse PAR

**Figure 4:**
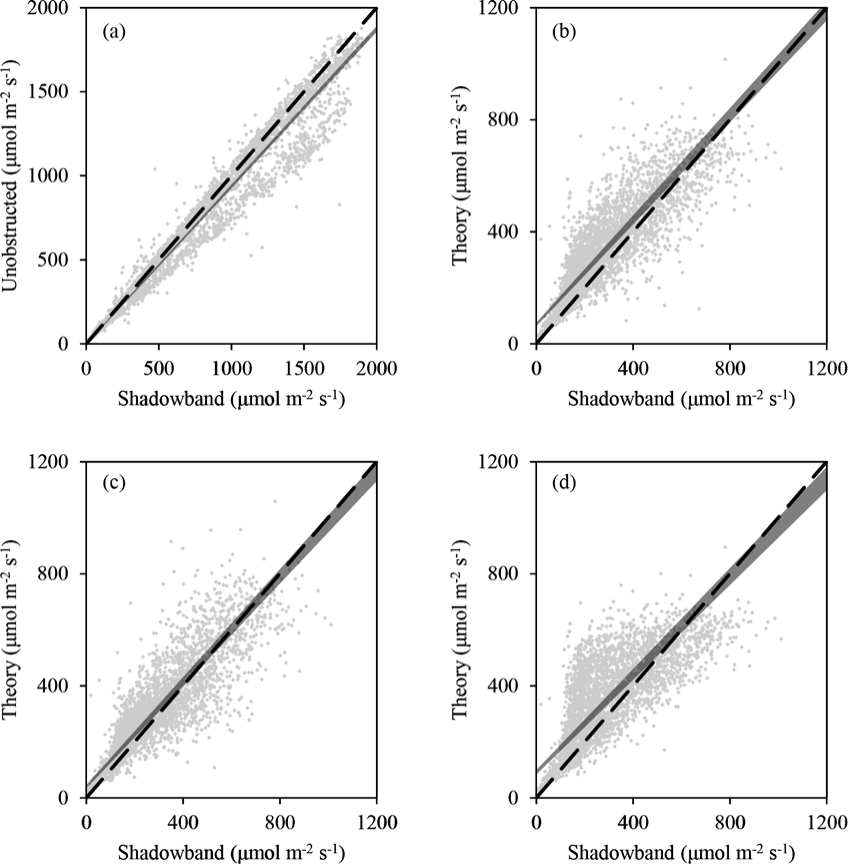
Scatter plots comparing PAR values measured by shadowband system with other accepted methods of quantifying PAR. Dashed line represents ideal one-to-one relationship and grey wedge represents the 95% confidence interval computed using Deming regressions. a) PAR_T_ vs. unobstructed quantum sensor b) PAR_D_ vs. Spitters c) PAR_D_ vs. Erbs d) PAR_D_vs. Jacovides

The regression of total incident PAR measured by the shadowband system against the measured values taken by the unobstructed quantum sensor was nearly one-to-one, with a slope that was significantly different than 1 but was still between 0.9 and 1 and an intercept that did not differ from 0 (Table 4). There were two distinct data patterns in this comparison (Fig. 4a): one group of points clustered around the ideal one-to-one line and a second group of points that showed a slightly higher bias for the measurements made by the shadowband. The authors have determined that this higher bias in the second group of points is likely due to drift in the accuracy of the readings from either one or both of the LI-190 quantum sensors. Almost all of this biased data was collected during the final two months of the experiment, August and September of 2012, and this period was beyond the suggested period for recalibration of the sensors. When the analysis was run without these data the mean slope improved to 0.97 while the intercept differed significantly from 0 (mean = 10.73).

**Table 4:** Statistical analysis of Deming regression of shadowband measurements against empirically derived and measured data.

**Source of Comparison**	**Slope (95% CI)**	**Intercept (95% CI)**
*Measured total PAR*		
Clear PAR sensor	0.94 (0.93, 0.943)	0.86 (-3.242, 4.969)
*Empirical diffuse*		
Spitters et al. 1986	0.93 (0.909, 0.957)	70.12 (63.858, 76.372)
Erbs et al. 1982	0.94 (0.914, 0.963)	40.42 (34.331, 46.511)
Jacovides et al. 2010	0.87 (0.845, 0.901)	92.23 (84.321, 100.132)

When comparing the theoretical diffuse PAR models to the measurements of diffuse PAR made by the shadowband system all slopes and intercepts differed significantly from the ideal one-to-one values of 1 and 0, respectively (Table 4). Spitters et al. [23] (Fig. 4b) and Erbs et al. [27] (Fig. 4c) produced the best agreement with observed data collected by the shadowband system with regression slopes between 0.9 and 1 and relatively smaller deviations from the ideal intercept of 0. The regression between observed data and the approach of Jacovides et al. [12] (Fig. 4d) yielded a slope that deviated the furthest from a 1:1 line (0.87) and an intercept that deviated the most from the ideal of 0 (92.23). While the empirical approach of Jacovides et al. [12] agreed relatively well with our observed data at low PAR values, it exhibited a high bias for a range of mid PAR values and a low bias for the highest PAR values (Fig. 4d).

**Figure 5:**
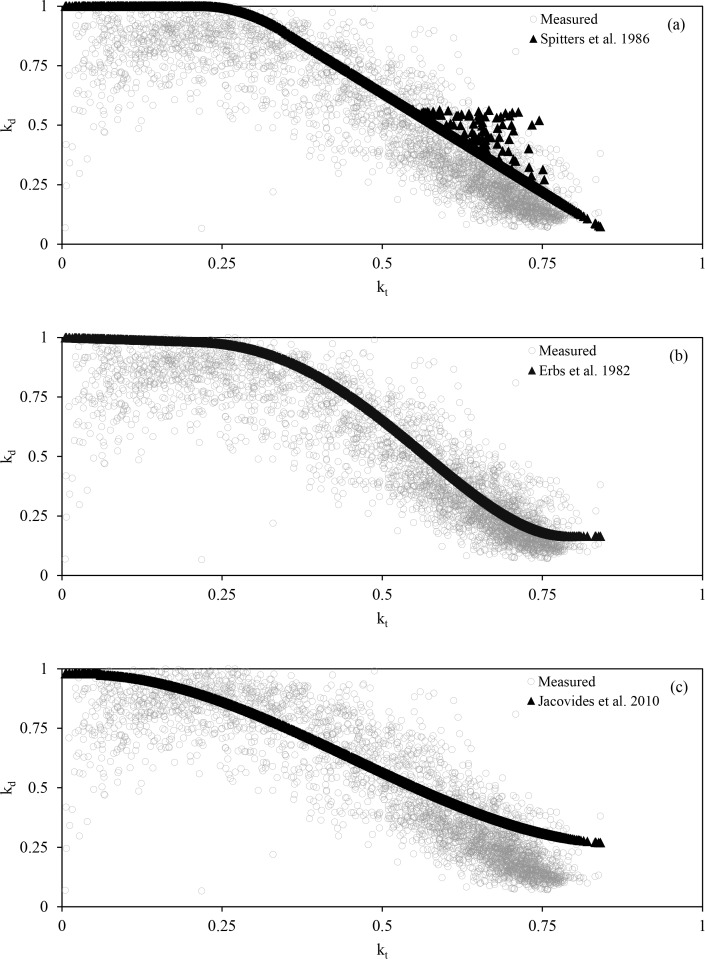
Relationship between clearness index (k_t_) and ratio of diffuse-to-global radiation (k_d_) for observed values plus 3 modeling approaches. a) Spitters et al. 1986 b) Erbs et al. 1982 c) Jacovides et al. 2010

On average, the percentages of PAR_D_ predicted from the three empirical approaches we studied were higher than the values measured by the shadow band system (Table 5; Fig. 4). None of the empirical approaches studied predicted a decrease in diffuse percentage at low k_t_ values that were observed at our site, which we verified to be occurring during morning and evening hours on approximately 45 days that had observed suspended moisture (e.g. foggy) or atmospheric particulates (e.g. hazy). These hourly observations were collected 20km south of the research site at the Dane County Regional Airport, Madison, WI (http://www.ncdc.noaa.gov/). A significant fraction of the measured data were collected when k_t_ values were in a range of 0.5 and 0.85, meaning that differences between the three empirical relationships studied and measured data in this range of k_t_ values greatly affected the overall model agreement. This is most apparent with the approach of Jacovides et al. [12] (Fig. 5), where many of the initial predicted values fit the measured data well, but the overall fit of the model was the worst of the three approaches. The approach of Spitters et al. [23] uses additional regression parameters (R and K) that appear to enhance replication of some natural variability that exists in the observed data, but only for higher k_t_ values and the additions only led to an increase in k_d_ values.

**Table 5:** Statistical analysis of the average difference between empirically derived diffuse percentages and measured values.

**Source of empirical relationship**	**Mean Difference**	**Std Dev**	**Pr > |t|**
Spitters et al. 1986	0.101	0.119	< 0.0001
Erbs et al. 1982	0.073	0.125	< 0.0001
Jacovides et al. 2010	0.076	0.128	< 0.0001

## Discussion

### 4.1. Shadowband system measures PAR_T_ with high confidence

The strong, almost one-to-one relationship between the two compared PAR_T_ values shows that the measurements of PAR_D_ and PAR_M_ made by the shadowband system led to an accurate quantification of PAR_T_. Deviations from the ideal one-to-one relationship could potentially be due to only taking 12 minimum (i.e., diffuse) measurements over the averaging period of one hour, leading to improper quantification of PAR_D_ over that period. This would then lead to error in the estimation of PAR_T_. These 12 minimum measurements are also sensitive to noise spikes, where an abnormal drop during the shaded measurement period could lead to a significant underestimation of diffuse PAR and negatively influence the accuracy of subsequent calculations. Independent drift in the sensor readings of the two LI-190s could also have led to a deviation in the one-to-one relationship. However, given the strong agreement between the two PAR_T_ values, it was concluded that this level of error was acceptable; the drift in the sensor readings was likely minimal other than at the end of the experimental period and the number of minimum diffuse PAR measurements (12 per hour) was appropriate.

### 4.2. Shadowband system data elucidates problems with applying generalized models at individual research sites

With two out of the three empirical approaches for estimating PAR components that we analyzed comparing reasonably well with our observed data, the shadowband system produces acceptable values over a range of atmospheric conditions. However, the empirical models were biased towards predicting higher fractions of diffuse radiation compared to our measurement approach. Model errors should be expected because the theoretical relationships are designed to estimate mean values over long time periods and are not necessarily designed to predict high frequency variation in PAR, leading to foreseeable differences between predicted and observed hourly PAR_D_. Also, the piecewise data fitting strategies used for the empirical approaches require a continuous model through segmentation points and so are limited by assumptions made in designing the model, such as setting a high percentage of PAR_D_ at low k_t_ values. Furthermore, these empirical models were derived from data originating from locations other than our research site (Jacovides from the slopes of Mt. Hymettus in Athens; Erbs from four United States cities outside of the Midwest; and Spitters from the Netherlands [28]). These location differences would lead to variations in important environmental conditions, e.g. relative sunshine duration, water content of the atmosphere and cloud type [23], which influence the measured relationship between diffuse percentages and atmospheric transmission. The Athens research site would be exposed to significantly different types of seasonal cloud cover than the research site stemming from its Mediterranean climate. The Erbs data, while captured in the United States, is associated with major cities that vary significantly in latitude and longitude. The proximity with cities would expose radiation measurements to different particulate matter than what would be seen at remote locations like the research site presented here, and the differences in location would lead to differences in sunshine duration and the length of the atmospheric radiation path. The theory presented by Spitters et al. [23] is used often in ecosystem modeling efforts [29] but significant differences in the location of their data sets compared to the measurement site presented here leads to potentially significant differences in environmental conditions; these differences can thereby contribute to a diminished performance of the empirical model when applied at specific locations.

All three of the empirical modeling approaches used here for comparison to observational data used a LI-200 pyranometer (LI-COR, Inc.) to obtain a measurement of k_t_ that was then used to predict the PAR diffuse radiation fraction. As mentioned before, this particular sensor has a less than ideal response across the 400–1100nm wavelength band, especially under non-full sunlight conditions [30], and only has a 25% to 75% relative response to irradiance in the 400–700nm range that we were most interested in. While this sensor has compared well against other total radiation sensors under ideal conditions and presents a much less expensive option for solar radiation measurements, the potential errors made by this sensor could have led to errors in the prediction of diffuse radiation values by the three empirical approaches. In contrast, Jacovides et al. [12] provides an example of empirical equations that were derived using measurements made by a Kipp & Zonen model CM11 pyranometer (Delft, The Netherlands), which has a superior response to incoming radiation compared to the LI-200, but is much more expensive. However, the improved quality of these measurements could lead to an improved understanding of diffuse radiation dynamics even though this theory did not transfer as well to this study.

These comparisons suggest generalized relationships between diffuse percentages and atmospheric transmission can be derived from local measurements; however, these empirical models are unable to capture the influence of short-term, site specific environmental characteristics on these relationships [31, 32] and site specific data will always improve the accuracy of radiation modeling for a location. Scientists need to be aware of limitations in these empirical modeling approaches as well as limitations of instrumentation that is used to build models that characterize components of PAR for a range of atmospheric conditions.

### 4.3. Potential system errors

The majority of measurement errors that drove the removal of 19% of the available data occurred either when the shadowband was not rotating or when the percentage of PAR_D_ was close to 100%. Without shadowband rotation, the measurement of PAR_D_ will be close to maximum incident values, even on clear days. With diffuse radiation percentages close to 100% the calculated PAR_D_ can be higher than the calculated PAR_T_ due to 1) the +/- 5% error inherent to the quantum sensor calibration [33] and 2) the use of a 15% correction factor for radiation blockage by the shadowband during the measurement. Both of these errors can be checked for by comparing hourly PAR_T_ and PAR_D_ values and these issues can be addressed ahead of time by maintaining a consistent power source to the system and by keeping the calibration of the quantum sensor up to date.

### 4.4. Equipment cost and required maintenance is reduced compared to other available systems

The final cost of all necessary parts for the presented shadowband apparatus was ~$4,700 USD. This price includes an upgrade for the solar panel, from an SP70 to an SP90 (90 W), because the SP70 was longer carried by Campbell Scientific. This final price places the cost of the proposed system below all of the devices described in the introduction and about 50% lower than any of the instruments that are intended to be setup and left for long periods of time with periodic adjustments. While the presented system requires some metal fabrication, it is believed that the added workload is minimal and that the lower input costs more than make up for the inconvenience. Furthermore, the use of an onsite AC power source would reduce the system cost by approximately $1000. Other components of the system may also be interchanged with other commercially available equipment to potentially save on cost. For example, the CR1000 is a high end datalogger capable of far more than what is presented here and replacing this part with a cheaper datalogger from Campbell Scientific or another distributor could significantly reduce the overall equipment cost.

### 4.5 System portability and installation

Because the system can be broken down into its component parts (e.g. logger enclosure, solar panel, tripod, etc.) with relative ease, it proved relatively easy for 2 people to take down and setup the system. The only pieces with considerable weight are the tripod (15 kg), enclosure (7.7 kg), battery (25.9 kg) and solar panel (7.7 kg). The tripod (145 cm when folded up) is the only piece that requires extra room when being transported. If the power supply for the system was changed from the solar panel to an AC source, this would remove multiple pieces of equipment (solar panel and rechargeable battery) that would make the system even more portable.

While the components and design of the shadowband motor area and sensor stands is designed to allow leveling, it is important to have a base that is as level as possible. To this extent, the site for the tripod was leveled as accurately as possible before installation and cinder blocks were buried to provide a solid base for the tripod. As an alternative, the tripod could be replaced with a smaller tripod or an instrumentation tower as long as the base was level and the height of the shadowband installation was above all objects that could block radiation measurements. After installation, the leveling should be checked after about a week to make sure settling has not greatly shifted the setup; typically the system could be checked for leveling once a month or less after that. Recalibration of specific instruments (e.g. radiation sensors) will be dependent upon equipment used and company suggestions.

### 4.6 Additional system uses

While the presented system was designed for ecological studies that often require measurements of photosynthetically active radiation (PAR) on a horizontal plane parallel to the surface of the earth (e.g., a leaf or plant canopy), there are a few potential adjustments or additions to the system that would allow it to be used in a wider range of applications. For applications that require measurements of total incoming solar radiation, simply replacing the PAR sensor with a sensor that measures total solar radiation, such as the LI-200, would accomplish this goal but as mentioned before sensor limitations would have to be considered and use of a different style of sensor may require changes to shadowband dimensions and values used in the theory section. Another option for computing total solar radiation from the systems base measurements is to use assumed values of PAR percentages of total incoming solar radiation and doing the back calculation to acquire a total incoming value. This approach will be laden with the obvious errors of using assumed conversion values for what is a variable value based on atmospheric conditions.

While the system cannot directly measure direct normal irradiation (DNI), the measurement of total incoming solar radiation perpendicular to direct beam radiation, it does measure enough variables that calculation of DNI would be possible. If the PAR sensor was replaced by a sensor that measures total solar radiation and independent calculations of solar zenith angles were made, such as those used in the calculation of diffuse radiation for the theories used in this paper, DNI could be calculated using the cosign relationship between beam radiation on a horizontal plane and solar zenith angles. While direct measurements of DNI would be more accurate, the type of measurement described here would not require installing a sun tracking system which would greatly increase system complexity and cost.

## Conclusions

**Figure 6:**
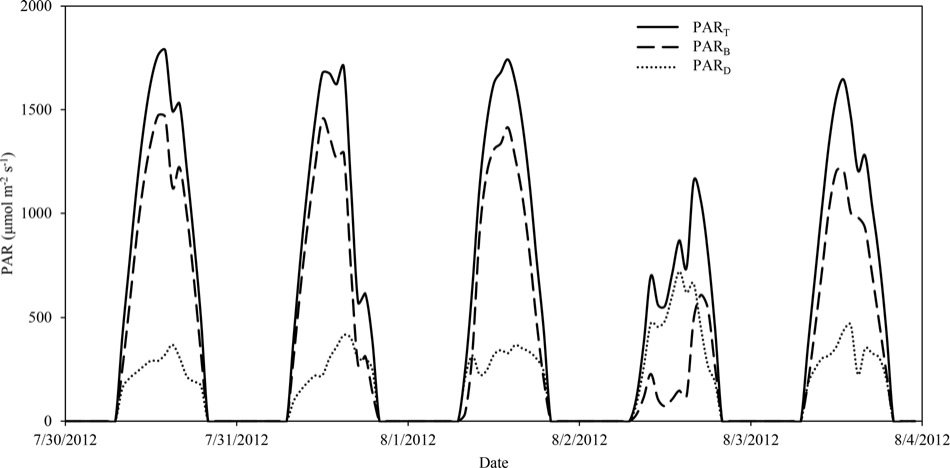
Example time series of radiation measurements.

The data produced using the concept of a rotating shadowband system (Fig. 6) indicates that this type of apparatus could be counted on to reliably collect high quality data at field research sites. Deming regressions between shadowband produced data and other accepted methods of quantifying incident photosynthetically active radiation indicate that the system agrees very well with other sensors taking onsite measurements and captures variability at an hourly time scale that would be missed by using widely accepted theoretical relationships. Given the simplicity of both system structure and theory, we believe the shadowband apparatus would be an effective fit for many different research endeavors and could be used to expand data collection of total incident photosynthetically active radiation and its components to a wider variety of remote areas. A few options for implementation of this apparatus include finding a source of continuous AC power at the research site and using other component parts that may be more familiar to the user than parts presented here. And finally, consideration should be given to adding two full spectrum pyranometers: one to take unobstructed measurements and one to be used with a second shadowband system. This will allow for a better understanding of the relationship between the clearness index of the atmosphere and the ratio of the diffuse-to-global solar radiation, which in turn will aid in extrapolating results beyond the measurement site itself.
